# Conduction Band Edge Energy Profile Probed by Hall Offset Voltage in InGaZnO Thin Films

**DOI:** 10.3390/mi11090822

**Published:** 2020-08-30

**Authors:** Hyo-Jun Joo, Dae-Hwan Kim, Hyun-Seok Cha, Sang-Hun Song

**Affiliations:** School of Electrical and Electronics Engineering, Chung-Ang University, Seoul 06974, Korea; hyojunjun@naver.com (H.-J.J.); pccdhkim@naver.com (D.-H.K.); ckgustjr0803@naver.com (H.-S.C.)

**Keywords:** n-channel InGaZnO (IGZO), Hall offset voltage, conduction band edge energy profile, percolation conduction

## Abstract

We measured and analyzed the Hall offset voltages in InGaZnO thin-film transistors. The Hall offset voltages were found to decrease monotonously as the electron densities increased. We attributed the magnitude of the offset voltage to the misalignment in the longitudinal distance between the probing points and the electron density to Fermi energy of the two-dimensional electron system, which was verified by the coincidence of the Hall voltage with the perpendicular magnetic field in the tilted magnetic field. From these results, we deduced the combined conduction band edge energy profiles from the Hall offset voltages with the electron density variations for three samples with different threshold voltages. The extracted combined conduction band edge varied by a few tens of meV over a longitudinal distance of a few tenths of µm. This result is in good agreement with the value obtained from the analysis of percolation conduction.

## 1. Introduction

Oxide semiconductors are used in various applications, including flat-panel displays [1,2], optical sensors [3,4], and solar cells [5,6], owing to their excellent electrical and optical properties. Among them, the amorphous-InGaZnO (a-IGZO) thin-film transistors (TFTs) have attracted considerable attention for applications in flat-panel displays thanks to their high electrical performances [7,8,9,10,11,12] and manufacturability over a large area [13]. For this particular material, the conduction band minimum was originated from the isotropic s-orbitals of the cations, resulting in a small effective mass and high electron mobility for superior electrical properties, even in amorphous phase. In addition, its lack of crystallinity helps rapid improvements in the bending performances of the a-IGZO TFTs [14,15], which are expected to pave the way to potential applications in flexible electronics on plastic substrates. Field-effect mobility as high as 160 cm^2^/Vs was reported by reducing defects with a strong capping layer at the back interface [12]. However, the current conduction mechanisms in a-IGZO TFTs have to be understood in depth to further improve their electrical characteristics for future applications in faster and larger displays. For this purpose, the fundamental carrier-transport properties of a-IGZO thin films were extensively studied by Hall measurement [16] and electrical transport measurements [17,18], which were interpreted as trap-limited conduction in low-gate voltage operations and percolation conduction at high-gate voltages. Furthermore, the electronic band structures of a-IGZO were deduced. In these transport studies, two of the key parameters were the carrier density and the mobility, which are generally measured as functions of various parameters in the measurements. Therefore, Hall measurements from which these two parameters can be determined simultaneously, are of pivotal importance in semiconductor transport studies. However, it is very challenging to perform Hall measurements on low mobility [19].

The Hall offset voltage, i.e., the output voltage signal perpendicular to the current direction in the absence of magnetic induction, should be zero in perfectly aligned Hall probing points. However, non-zero Hall offset voltages [20] are common in real devices, especially devices with van der Pauw geometry where the probe misalignments are quite frequent. In such cases, if the measured Hall data are not carefully analyzed, the devices will be characterized incorrectly.

In this study, we carefully analyzed the Hall offset voltages in Hall bar-patterned a-IGZO TFT samples in magnetic fields with both negative and positive polarity. The Hall offset voltages were measured with an AC Hall setup to obtain noise-reduced results. Despite the Hall bar geometry, which was chosen to avoid the complexities arising from the misalignments of the Hall probes, Hall offset voltages were still present in our measurements. We interpreted the measured Hall offset voltage as the unintentional misalignment distance and the electron density as the Fermi energy level. We constructed combined conduction band edge energy profiles for three samples with different threshold voltages by measuring the Hall offset voltages as a function of the two-dimensional electron density. The extracted conduction band edge energy variation of a few tens of meV was in good agreement with the extracted average potential barrier height from percolation conduction model [16].

## 2. Experimental Procedure

**Figure 1:**
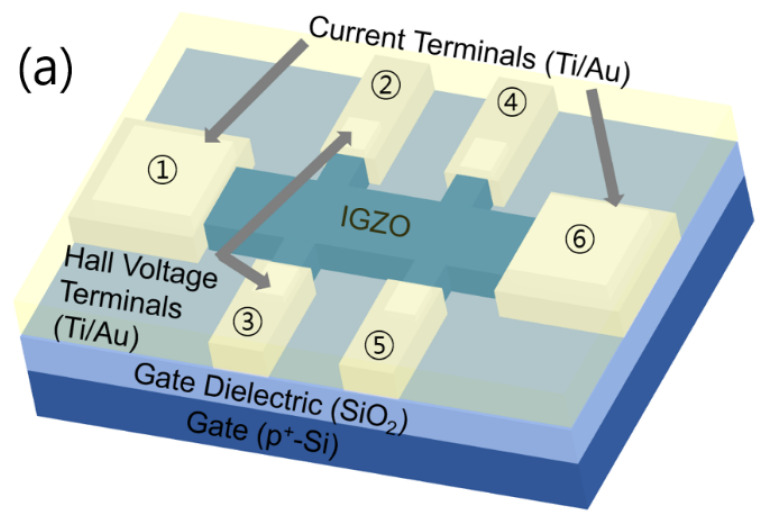

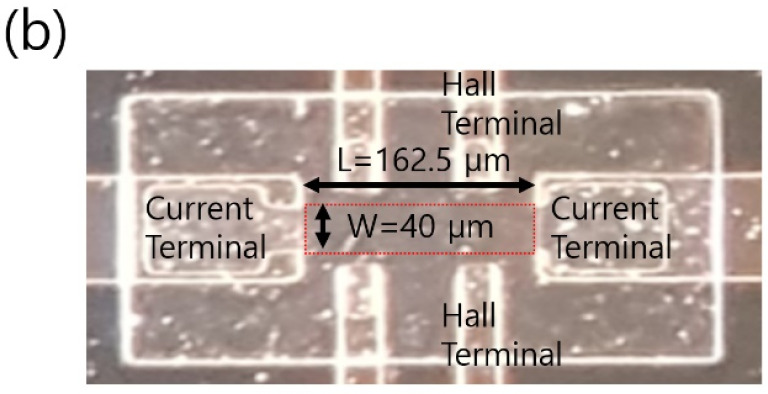
(**a**) Schematic of the a-IGZO Hall bar TFT without the top SU-8 passivation layer. The electrode terminals are labelled by numbers from 1 to 6. (**b**) Optical image of the fabricated a-IGZO TFT with the top passivation layer.

Figure 1a shows a schematic of the fabricated a-IGZO TFT Hall bar device without the top SU-8 passivation layer, which is generally used to reduce the instability associated with oxygen adsorption [21,22]. The n-type a-IGZO TFTs were fabricated on top of a 100-nm-thick thermal SiO_2_ layer grown on a heavily doped p-type Si substrate used as a gate electrode in the measurements. A 50-nm-thick a-IGZO channel layer was deposited on the cleaned SiO_2_ layer by RF magnetron sputtering at room temperature. Hall bar patterning was performed through a liftoff process and, subsequently, thermal annealing was performed at 450 °C for 30 min in air. A nonmagnetic 70-nm-thick Ti layer and a 30-nm-thick Au layer were deposited using e-beam evaporation and were patterned by liftoff to serve as the electrodes of the a-IGZO TFTs. Finally, a 2-μm-thick SU-8 layer was formed as a passivation layer using a spin-coating process. Figure 1b shows an optical image of the fabricated a-IGZO TFT with the passivation layer. Two current terminals were placed horizontally and two pairs of Hall and longitudinal voltage terminals were placed vertically. The vertical Hall terminals were lithographically aligned to prevent unintentional misalignment and the center spacing between the two horizontal longitudinal terminals was 87.5 μm. The electrode terminals were labelled by numbers from 1 to 6. The channel width (*W*) of the device was 40 μm and the length (*L*) was 162.5 μm.

## 3. Results and Discussion

**Figure 2:**
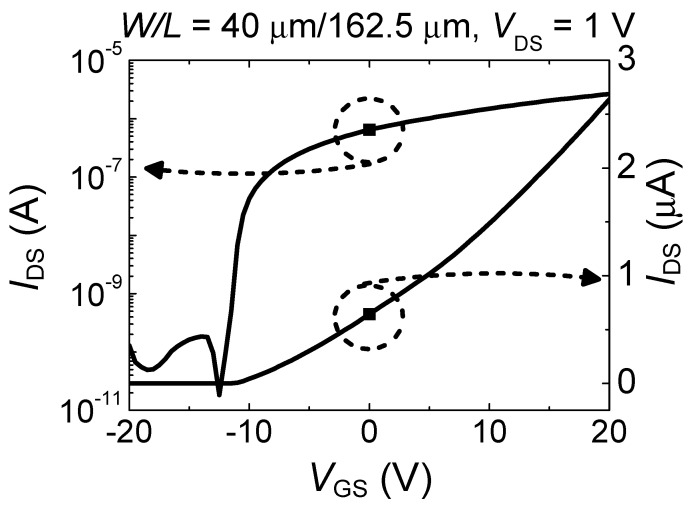
Transfer curve of the a-IGZO Hall bar-patterned TFT.

Figure 2 shows the drain current *I*_DS_ as a function of the gate-source voltage *V*_GS_, i.e., the transfer curve, measured in our device. The *I*_DS_ is reported in logarithmic scale to clearly show the on/off characteristics, and the *V*_GS_ is in linear scale to deduce the threshold voltage. Here, we used two current terminals as source/drain terminals (1 and 6) and the p+ Si substrate as a gate terminal. The transfer curve was measured at drain-source voltage (*V*_DS_) of 1 V while sweeping *V*_GS_ from −20 to 20 V to fully show the on/off characteristics. The values of field-effect mobility (*μ*_FE_), the on/off current ratio, threshold voltage (*V*_TH_), and subthreshold swing were measured to be 14.56 cm^2^/Vs, 1.76 × 10^5^, −8.69 V, and 0.42 V/decade, respectively. The value of *μ*_FE_ was calculated from the maximum transconductance in the linear regime (*V*_DS_ = 1 V), and that of *V*_TH_ was extracted by extrapolating a straight line in *I*_DS_-*V*_GS_ plot on the *V*_GS_ axis. These values (extracted from the transfer curve) are in good agreement with those reported in the literature [23].

**Figure 3:**
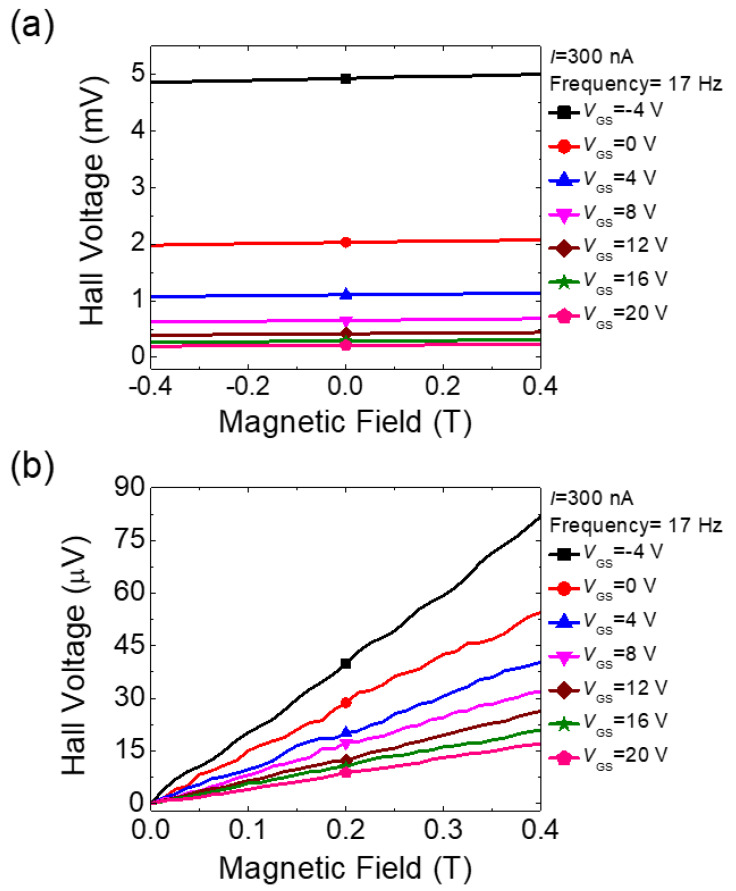
(**a**) Hall voltages as a function of the magnetic field with various gate-source voltages *V*_GS_ (−4, 0, 4, 8, 12, 16, and 20 V); a 17-Hz, 300-nA sinusoidal current was used. (**b**) Hall voltages with offset corrected by applying magnetic field reversal measurement data.

Figure 3a depicts the plots of the Hall voltages with *V*_GS_ ranging from −4 to 20 V in the a-IGZO Hall device as a function of the applied bipolar magnetic field. We adopted a low-frequency ac Hall measurement to achieve the reasonably high signal-to-noise ratio for later analysis. A 17-Hz sinusoidal ac current (*I*) of 300 nA was applied between the current terminals (1 and 6). The magnetic field (*B*) from −0.4 to 0.4 Tesla was applied perpendicularly to the Hall device, and the Hall voltage (*V*_H_) was measured between the Hall probe terminals (2 and 3) while sweeping *B*. From the figure, it can be seen that the measured Hall voltages between the two intentionally aligned Hall probes linearly increased as *B* increased from −0.4 T to +0.4 T, following classical Hall theory. However, non-zero offset values were observed at *B* = 0 T, which is contrary to the classical Hall theory [24]. In addition, the offset voltage decreased monotonously with the gate voltage *V*_GS_. The offset was attributed mainly to the unintentional misalignment of the two Hall voltage probes [25,26,27]. This decrease will be analyzed later after extracting the two key parameters of the sample, the electron density and the mobility. To extract electron densities from the measured Hall voltages, the offsets need to be corrected with the measurements obtained when reversing the direction of current flow and magnetic field [28]. The current direction reversal was automatically taken care of in the ac Hall measurements. For the magnetic field reversal measurements, bipolar magnetic field sweeps were performed, and the results are illustrated in Figure 3a. The measured Hall voltages shown in Figure 3a are corrected by taking the half of the value obtained by subtracting the Hall voltage at negative *B* from the one at positive *B*. The results are shown in Figure 3b. It is worth noting that the Hall offset voltage was in the range of mV and the offset-corrected Hall voltage was in the range of tens of μV. The Hall voltage was approximately two orders of magnitude smaller than the Hall offset voltage. Thus, achieving a reasonably high signal-to-voltage ratio is critical in these measurements. The offset-corrected Hall voltages increased linearly with the magnetic field, indicating that there existed a single channel in the device [29]. The electron density in the device can be calculated from the slope of the Hall voltage vs. magnetic field curve, and the mobility can be calculated from the measured resistance between two longitudinal voltage probes (3 and 5).

**Figure 4:**
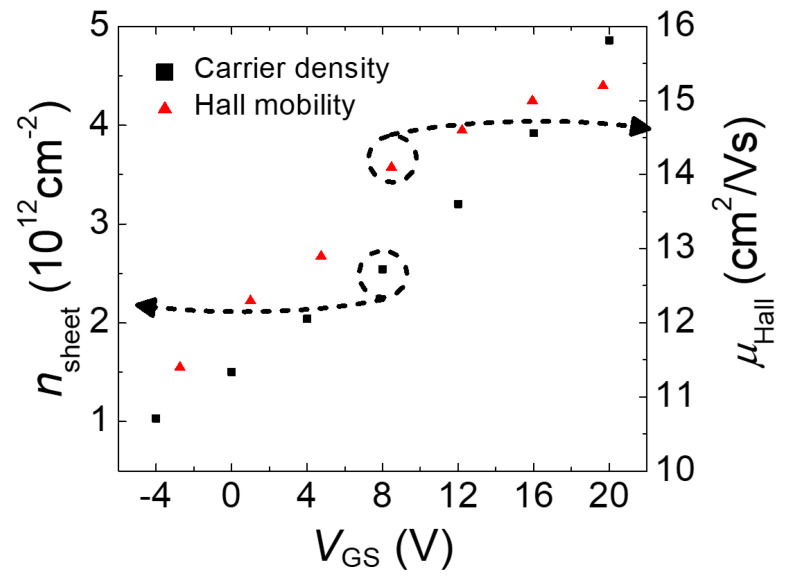
Electron density (*n*_sheet_) and Hall mobility (*µ*_Hall_) as a function of the gate voltage *V*_GS_.

**Figure 5:**
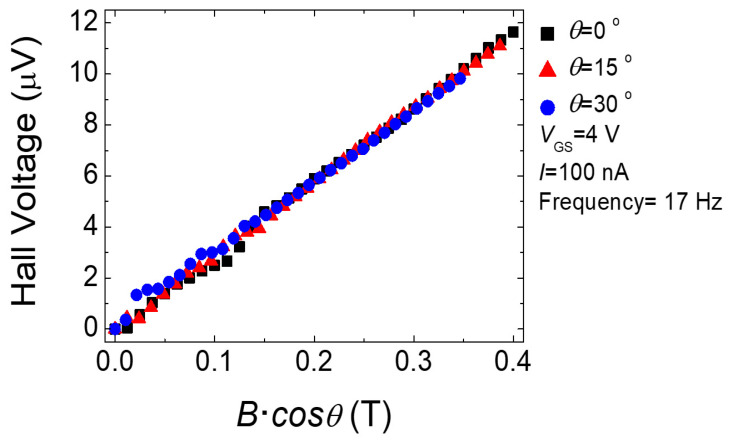
Offset-corrected Hall voltages at a gate voltage *V*_GS_ of 4 V and current level *I* of 100 nA as a function of the perpendicular magnetic field at tilting angles of 0°, 15°, and 30°.

Figure 4 shows a plot of the electron density (*n*_sheet_) and Hall mobility (*μ*_Hall_) as a function of the gate voltage *V*_GS_. It can be seen that the areal electron density increased linearly as V_GS_ increased, indicating a capacitively induced channel. The monotonous increase in *μ*_Hall_ as a function of the electron density is a typical behavior observed in a-IGZO TFT [17]. To verify the dimensionality of the electrons, tilted magnetic field measurements at tilting angles of 0°, 15°, and 30° were performed at *V*_GS_ = 4 V, at which reasonably high Hall voltages were expected. A smaller current *I* = 100 nA was used to minimize perturbations. Figure 5 shows the results of the offset-corrected Hall voltages at the perpendicularly applied magnetic fields. The coincidence of the Hall voltages with respect to the perpendicular *B*’s confirms that the electrons were confined in two dimensions [30].

**Figure 6:**
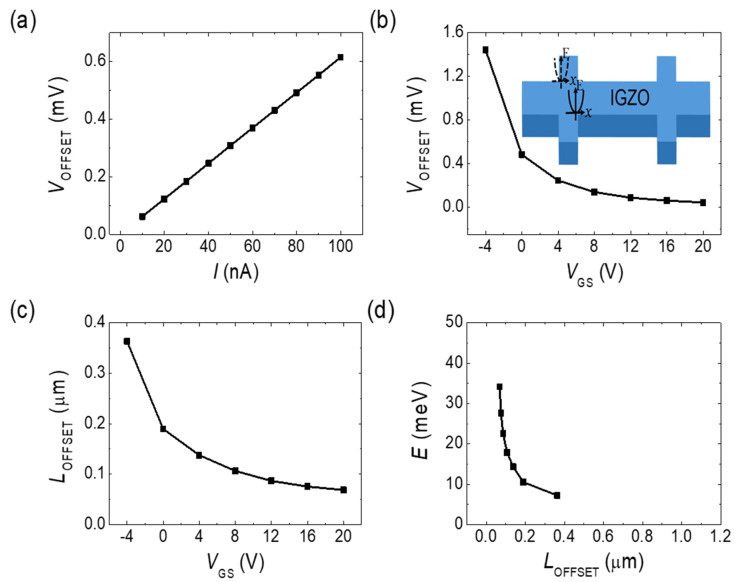
Offset voltage as a function of the (**a**) applied current and (**b**) gate voltage *V*_GS_. The inset shows a schematic of the device with the possible probing point conduction band energy profiles. (**c**) Offset length versus *V*_GS_. The offset length was converted from the offset voltage using Equation (1). (**d**) Fermi energy versus offset length. The Fermi energy was calculated by dividing the electron density by the 2 dimensional electron density of states. This plot is interpreted as the combined conduction band edge energy profile.

Figure 6 shows the Hall offset voltage as a function of *V*_GS_. In Figure 6a, the measured offset voltage clearly shows a linear dependence on the applied current, indicating a resistive behavior. As mentioned earlier, if the offset voltage is assumed to be induced from the misalignment of the Hall probes, it can be interpreted as the voltage across the resistor in the current direction between the unintentionally misaligned probing points. Figure 6b shows a plot of the measured Hall offset voltage versus the applied *V*_GS_, and the inset shows the possible locations and energy profiles of the probing points. This was attributed to the misalignment of longitudinal length, resulting in the decrease in the Hall offset voltage as the electron density increases. This offset length *L*_OFFSET_ can be calculated from the Hall offset voltage *V*_OFFSET_, longitudinal voltage *V*_3,5_, and length *L*_3,5_ between the two longitudinal probes as follows:

(1) $$L_{\mathrm{OFFSET}}=L_{3,5}\frac{V_{\mathrm{OFFSET}}}{V_{3,5}}$$

Figure 6c show a plot of *L*_OFFSET_ as a function of *V*_GS_. Similar results can be obtained if the offset length is calculated from the geometry and the *V*_GS_-dependent conductivity value. From Figure 6c, it can be seen that *L*_OFFSET_ decreased monotonously as *V*_GS_ increased and, thus, the electron density increased. The increase of the two-dimensional electron density will induce a linear increase in the Fermi energy because the density of states is constant in two dimensions. The electron density can then be converted into the Fermi energy by simply dividing it by the two-dimensional density of states m*/πћ^2^, where m* is the electron effective mass of 0.34 m_0_ [23] and ћ is the Planck’s constant. Figure 6d shows a plot of the calculated Fermi energy versus the offset length. The x and y axes are interchanged from Figure 6c to represent the energy profile. The Fermi energy varies by approximately tens of meV over a length of a few tens of μm. This energy range is in good agreement with the reported result of the average barrier height range obtained from the percolation conduction model [23].

In percolation conduction, the conduction band edge has potential energy fluctuations and results in a nonflat, corrugated energy surface. Electrons find and flow along low-lying constant energy paths, inducing a current. If percolation conduction is assumed in the sample with many voltage probes (as in our case), the low-lying energy probing point locations are expected to be randomly placed in the active area even though the probes are lithographically aligned. In the a-IGZO TFT Hall probes, the actual probing points are located at the lowest, filled conduction band, as shown in the inset of Figure 6b. Therefore, the offset length deduced from the Hall offset voltage can be interpreted as the longitudinal distance between these randomly located top and bottom Hall probing points. Furthermore, this length varies with the two-dimensional electron density. The measured offset length can then be interpreted as the distance between the facing edges of the Hall probing points at the Fermi energy. Therefore, by measuring the offset length as a function of the density, the combined conduction band profile from the facing edges was determined as shown in Figure 6d. For identical top and bottom profiles, the length would be halved for each profile.

**Figure 7:**
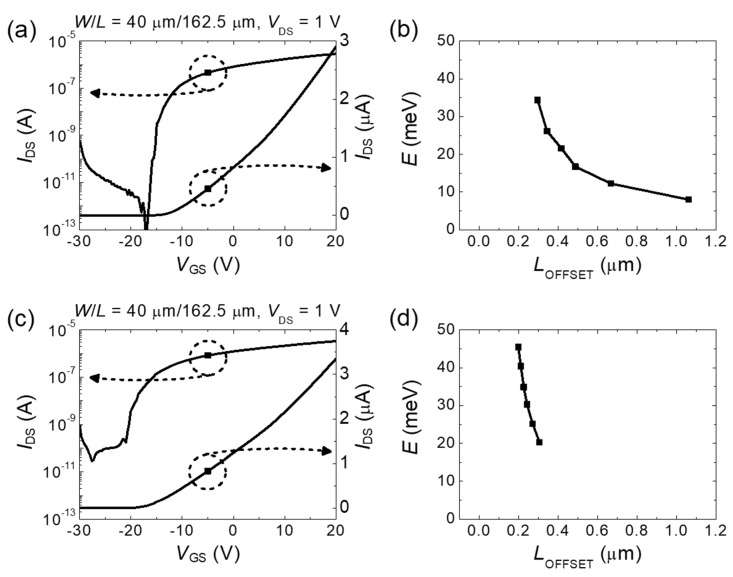
Combined conduction band edge energy profiles from two other samples. (**a**) Transfer curve of Sample B with *V*_TH_ = −10.01 V. (**b**) Deduced combined conduction band edge energy profile of Sample B. (**c**) Transfer curve of Sample C with *V*_TH_ = −15.82 V. (**d**) Deduced combined conduction band edge energy profile of Sample C.

Figure 7 shows the results from two other samples with different *V*_TH_. Figure 7a,c shows the transfer curves of samples B and C, respectively, with the same measurement conditions except for the *V*_GS_ sweep range. The *V*_GS_ sweep was extended to −30 V to show the full on/off characteristics due to different threshold voltages. Figure 7b,d shows the deduced combined conduction band edge profiles in samples B and C, respectively. The results reported in Figure 7 were obtained using threshold voltages of −10.01 and −15.82 V, respectively. The threshold voltage determines the range of the electron density for the same *V*_GS_ sweep. In particular, a lower threshold voltage leads to a higher electron density at the maximum *V*_GS_. Thus, the Fermi energy range of sample C can increase. It can be seen that the three samples measured showed similar profiles.

## 4. Conclusions

In this work, we systematically analyzed the Hall offset voltages of the two-dimensional electron system in three IGZO TFTs. The electrons were verified to be confined in two dimensions from the coincidence of the Hall voltages with respect to the perpendicularly applied magnetic field with tilting angles of 0°, 15°, and 30°. The Hall offset voltages decreased monotonously as the electron densities increased. We attributed the magnitude of the offset voltage to the misaligned longitudinal voltage between the probing points and the electron density to the Fermi energy. From these results, we deduced the combined conduction band profiles for the three samples with different threshold voltages. The extracted conduction band edge variation range of a few tens of meV is consistent with the value obtained from the analysis of percolation conduction [16].
